# Microbiological safety and pH stability of autoclaved pea protein-based beverages during short-term storage

**DOI:** 10.3389/fnut.2026.1795658

**Published:** 2026-05-19

**Authors:** Lasma Plocina, Ilze Beitane

**Affiliations:** Food Institute, Faculty of Agriculture and Food Technology, Latvia University of Life Sciences and Technologies, Jelgava, Latvia

**Keywords:** pea protein isolate, plant protein beverage, microbiological stability, thermal treatment, pH stability, thermal processing

## Abstract

Ensuring microbiological safety while maintaining moderate thermal treatment is a critical challenge in the development of plant–based protein beverages. This study evaluated the microbiological and pH stability of five pea protein isolate beverages with different fruit–and berry–based compositions. Beverages were subjected to thermal treatment in an autoclave at 105–115 °C for 5–20 min, followed by storage at 5 °C and 22 °C for 14 days. Immediately after thermal treatment, all beverages demonstrated high microbiological safety; however, storage stability varied depending on beverage composition, processing intensity, and storage temperature. Refrigerated storage at 5 °C maintained microbiological stability across all treatments, whereas room–temperature storage at 22 °C led to yeast and mold growth in certain beverage variants when lower–intensity treatments (105–110 °C) were applied. Autoclaving at 115 °C consistently ensured complete microbiological stability in all beverages. Throughout the study, pH values remained stable, reflecting high chemical stability. These results indicate that microbiological stability in pea protein isolate beverages is determined by the interplay of beverage composition, acidity, and thermal processing parameters. Careful selection of processing conditions allows the production of safe and stable plant–based beverages while minimizing thermal load and improving process efficiency.

## 1 Introduction

Pea protein isolate has attracted increasing attention as a promising plant–based protein source for the food industry, driven by its high nutritional value and its alignment with sustainability–oriented production systems aimed at improving resource efficiency and reducing greenhouse gas emissions (1). Peas (*Pisum sativum* L.) represent one of the most important legume crops in the European Union (EU), with a production volume of approximately 2.0 million tons in 2018, cultivated on more than 1.0 million hectares, primarily in France, Germany, Lithuania, Spain, and Romania. In recent years, the area under pea cultivation in the EU has expanded, supported by Common Agricultural Policy measures and the promotion of legumes within ecological focus areas (2).

This increasing production capacity supports the use of peas not only as a primary agricultural commodity but also as a raw material for higher value–added food ingredients. In parallel, global demand for plant–based protein ingredients have grown rapidly, driven by the expansion of plant–based food products and increasing consumer interest in sustainable protein sources (2). Consequently, the processing of peas into protein isolates has emerged as a key development pathway, enabling protein concentration and broader application in functional and alternative protein products (3).

Pea protein isolate is particularly valued for its high protein content (approximately 80–85%) and favorable functional properties, making it a key ingredient in plant–based product compositions (2). Although the production of pea protein isolate requires substantial raw material input approximately 28% isolate yield from peas containing about 24% protein – model–based assessments indicate that the expansion of pea and rapeseed cultivation to meet growing demand for plant–based foods would require only a limited share of EU arable land (2). This suggests that increased production of pea protein isolate can be achieved without significant pressure on agricultural land resources.

Moreover, pea cultivation offers several agronomic advantages, including a short growing season, the ability to fix atmospheric nitrogen, and relatively low water and mineral fertilizer requirements, all of which contribute to environmentally sustainable agricultural systems (4). Taken together, the scale of pea cultivation in Europe and these sustainability benefits highlight pea protein isolate as a strategically important ingredient for the development of sustainable, protein–rich food products (3).

From a nutritional perspective, pea protein isolate is distinguished as a nutritionally relevant plant-based protein source. Biological value (BV), expressed relative to whole egg protein as a reference standard (BV = 100%), has historically been used to compare protein quality. According to literature data, pea protein products are generally associated with BV values of approximately 65–70%, which are comparable to those reported for other commonly used plant protein ingredients such as soy protein (approximately 70–74%) and higher than those of cereal proteins, including wheat protein (approximately 55–60%) (5). In contrast, animal-derived proteins typically demonstrate higher biological values due to their more balanced essential amino acid composition and greater digestibility. For example, whey protein has been reported to exhibit BV values of approximately 100–104%, whole egg protein is defined as the reference protein (BV = 100%), and casein generally shows BV values in the range of approximately 75–80% (5). Importantly, controlled human data support the high digestibility and amino acid bioavailability of pea protein isolate: mean real ileal amino acid digestibility was reported as 93.6 ± 2.9% for pea protein isolate compared with 96.8 ± 1.0% for casein. Moreover, pea protein isolate achieved a DIAAS of 1.00, and net postprandial protein utilization was not significantly different from casein, indicating efficient utilization of absorbed amino acids in healthy adults (6).

The nutritional quality of pea protein isolate is largely determined by its essential amino acid profile. It is particularly rich in lysine, arginine, and leucine, amino acids that play key roles in muscle metabolism, tissue repair, and neurological function (7). Lysine, often a limiting amino acid in cereal–based diets, is present in relatively high concentrations in pea protein isolate and significantly enhances the overall quality of plant–based dietary protein. Adequate lysine intake supports protein synthesis, collagen formation, calcium absorption, and immune function, thereby improving the balance of essential amino acids in predominantly plant–based diets (8, 9). In addition, arginine contributes to nitric oxide synthesis and vascular regulation, while leucine activates the mTOR pathway, which is central to muscle protein synthesis and recovery processes (10). Unlike animal proteins, pea protein isolate contains no cholesterol and is low in saturated fat, which may contribute to improved lipid profiles and reduced cardiometabolic risk (11). While animal proteins need not be fully excluded from the diet, partial replacement with plant–based proteins are widely recognized as a health–promoting and environmentally sustainable strategy (12). A notable nutritional distinction between animal and plant proteins lies in methionine content; animal proteins are typically high in methionine, and excessive intake may promote homocysteine formation, which is associated with oxidative stress and increased cardiovascular risk (11). The lower methionine content of pea protein isolate may therefore support a more balanced intake of sulphur–containing amino acids. The nutritional aspects discussed above are based on literature data and are included to provide context for the relevance of pea protein isolate, whereas the present study focuses specifically on microbiological and pH stability.

Pea protein isolate is commonly produced via wet fractionation, a process that ensures high protein purity and favourable solubility while reducing starch and antinutrient content. These characteristics contribute to its technological stability and suitability for diverse food applications, particularly plant–based beverages. The functional properties of pea protein isolate, including solubility and structural stability, are strongly influenced by environmental pH. Solubility decreases markedly near the isoelectric point (approximately pH 4.5–5.0), where protein aggregation occurs, potentially compromising product homogeneity. Outside this range – particularly under strongly acidic conditions (pH < 4.0) or neutral to mildly alkaline environments – pea protein isolate retains superior solubility and functional performance, supporting its application in liquid food systems and thermal processing (13, 14).

By combining pea protein isolate with other plant–based components, it is possible to develop nutritionally balanced food products. From a compositional perspective, ingredients such as nut powders, soluble fibres (e.g., inulin), and micronutrients including vitamins and minerals are typically added in small quantities and generally do not exhibit strong acidic or basic properties. Nut powders are close to neutral in pH and are composed primarily of lipids, proteins, and dietary fibre, while inulin is a neutral, non–ionized carbohydrate that does not affect acid–base balance. Vitamins and minerals are added in micro–quantities that are insufficient to significantly influence product acidity.

In fruit–based beverages, overall pH is largely determined by organic acids naturally present in fruit juices, such as citric and malic acids, resulting in a strongly acidic environment (typically pH 2.5–4.0). Consequently, the incorporation of functional ingredients such as nut powders, inulin, and vitamins are not expected to substantially increase pH or compromise acidity, which is a critical factor for microbiological stability (15–17). pH is a key determinant of stability in plant–based beverages, as more acidic conditions (pH 4.0–4.5) limit microbial growth, extend shelf life, and enhance protein and colloidal stability by reducing protein aggregation and phase separation during storage (13).

Thermal treatment, particularly autoclaving, plays a central role in ensuring microbiological safety. Autoclaving at 105–115 °C for 5–20 min effectively inactivates microorganisms, including spores, while preserving nutritional quality and sensory attributes (13). The microbiological stability of plant–based protein beverages is governed by the interaction of multiple factors, including thermal processing parameters, pH, sugar content, minerals, vitamins, and the absence of preservatives. Due to high water activity and the presence of fermentable nutrients, such beverages are particularly susceptible to microbial growth if thermal inactivation is insufficient (18, 19).

Although organic acids such as ascorbic acid are frequently used to regulate pH and improve oxidative stability, they do not exert strong preservative effects and are insufficient to inhibit yeast and mold growth, especially in sugar–containing products (18, 20). Sugars serve as readily available energy sources for microorganisms, and yeasts may proliferate even at pH < 4.5, particularly during storage at ambient temperature (21, 22). In addition, minerals and vitamins may influence microbial metabolism and protein stability if microbiological control is inadequate (23).

The autoclaving temperature range of 105–115 °C is considered suitable for acidic and mildly acidic liquid foods (pH < 4.6), as it provides effective microbial inactivation without excessive protein denaturation associated with conventional sterilization above 120 °C. Lower temperatures may be insufficient to control heat–resistant microorganisms, while higher temperatures can impair protein solubility and sensory quality (13, 24). Storage at 5 °C and 22 °C enables evaluation of product stability under refrigerated and realistic consumer conditions, with a 14–day period commonly applied for early–stage stability assessment (22, 23).

Against this background, the aim of this study was to evaluate the effects of different autoclaving temperatures and processing times on the microbiological safety and pH stability of preservative–free pea protein isolate beverages. The findings contribute to the technological development of plant–based protein beverages and highlight the potential of pea protein isolate as a nutritionally relevant and technologically suitable ingredient for functional food applications.

## 2 Materials and methods

### 2.1 Compositional characteristics of ready–made pea protein isolate beverages

**Figure 1:**
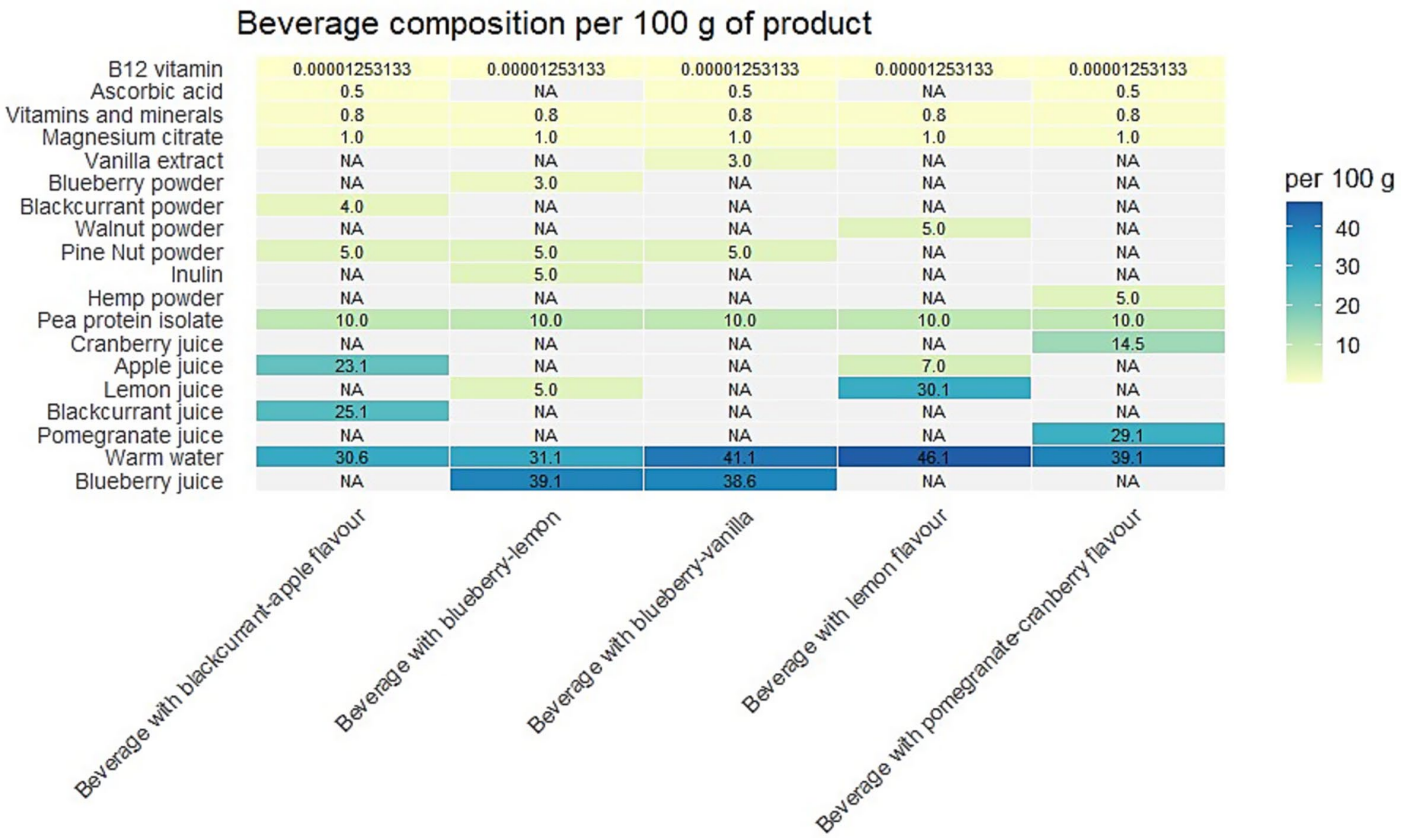
Composition of pea protein isolate beverages per 100 g of product. Heatmap representation of the distribution of key ingredients—pea protein isolate, water, fruit juice, powdered plant–based ingredients, vitamins, and minerals—across five beverage variants. The figure highlights compositional differences among the beverages, emphasizing variations in protein content, fruit–derived acidity, and functional additives.

The study evaluated five pea protein isolate beverages with different flavour profiles: lemon (LB), pomegranate–cranberry (PCB), blueberry–vanilla (BVB), blueberry–lemon (BLB), and blackcurrant–apple (BAB). The overall composition of the beverages is schematically presented in Figure 1. Ingredient selection was guided by predefined technological and sensory objectives rather than nutritional considerations alone.

Pea protein isolate was used as the primary ingredient, providing a high protein content and constituting the main component of the beverages. Nut and hemp powders were incorporated as sources of unsaturated fatty acids to improve the lipid profile. These ingredients were selected due to their content of omega–3 and omega–6 fatty acids, which contribute to a balanced dietary fat intake.

Fruit and berry juices, juice concentrates, or powders were used to establish the characteristic flavour profiles, natural colour, and acidic environment of the beverages, which are essential for both sensory quality and microbiological stability. Beverage acidity was primarily determined by organic acids naturally present in fruits, including citric and malic acids.

Inulin was added as a flavour–modulating component and as a source of soluble dietary fibre. Its inclusion helped to reduce potential off–flavours associated with plant proteins while increasing fibre content. As a non–ionized carbohydrate, inulin does not significantly affect beverage acidity and therefore does not alter the pH established by fruit–derived acids.

Vanilla extract was used as a natural flavouring agent to enhance sensory properties and harmonize the overall taste profile, particularly in beverages containing berry and plant–based components.

Ascorbic acid was added only to beverage variants with lower inherent acidity to adjust pH and limit lipid oxidation. In addition to its acidifying effect, ascorbic acid functioned as an antioxidant, contributing to the stabilization of fatty acids in beverages containing nut and seed powders.

A vitamin – mineral premix and magnesium citrate were included to providing essential micronutrients typically present in limited amounts in plant–based products. These components were added at levels that did not affect the pH or physicochemical stability of the beverages. All raw materials were stored according to the manufacturers’ recommended conditions and protected from direct sunlight and moisture prior to use. Beverages were prepared in a scientific laboratory at Latvia University of Life Sciences and Technologies under controlled room temperature conditions (18–22 °C). New, unused glass containers were used, as they are suitable for acidic food systems and do not interact with the product matrix.

All ingredients were weighed using laboratory scales (PS 1000, R2) with a precision of ±0.01 g. Drinking water preheated to approximately 72 °C was used as a solvent to facilitate dispersion of the ingredients. Components were added sequentially and manually mixed until a visually homogeneous dispersion was obtained.

After mixing, the containers were tightly sealed, and the prepared batches were immediately subjected to thermal treatment in an autoclave under predefined temperature and time conditions.

### 2.2 Autoclaving process and recording of temperature–pressure profiles

**Figure 2:**
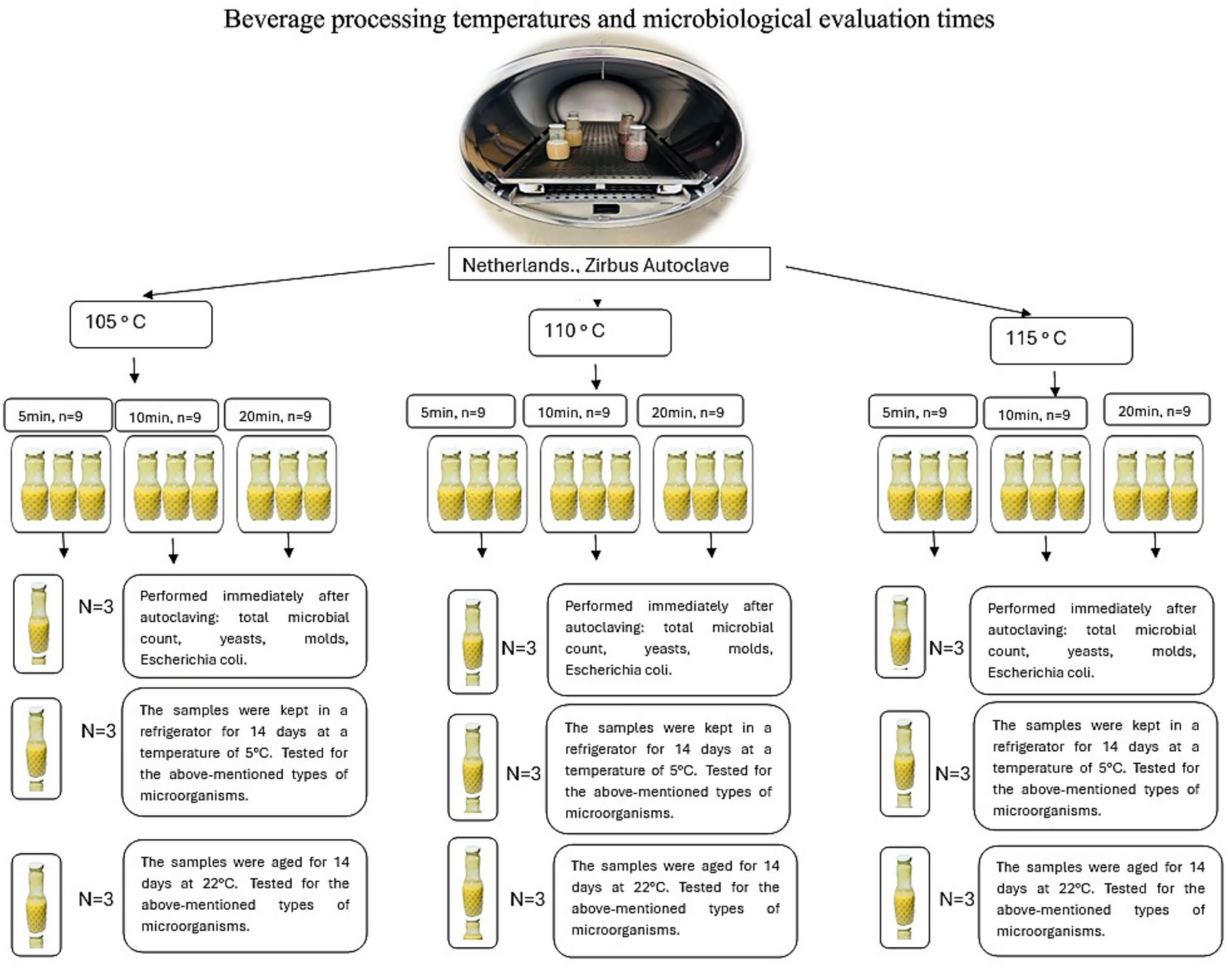
Thermal treatment regimens and microbiological evaluation schedule. Schematic illustration of the autoclaving conditions applied to pea protein isolate beverages (105, 110, and 115 °C with exposure times of 5, 10, and 20 min) and the corresponding time points for microbiological and pH analyses: immediately after processing and after 14 days of storage at 5 °C and 22 °C.

**Figure 3:**
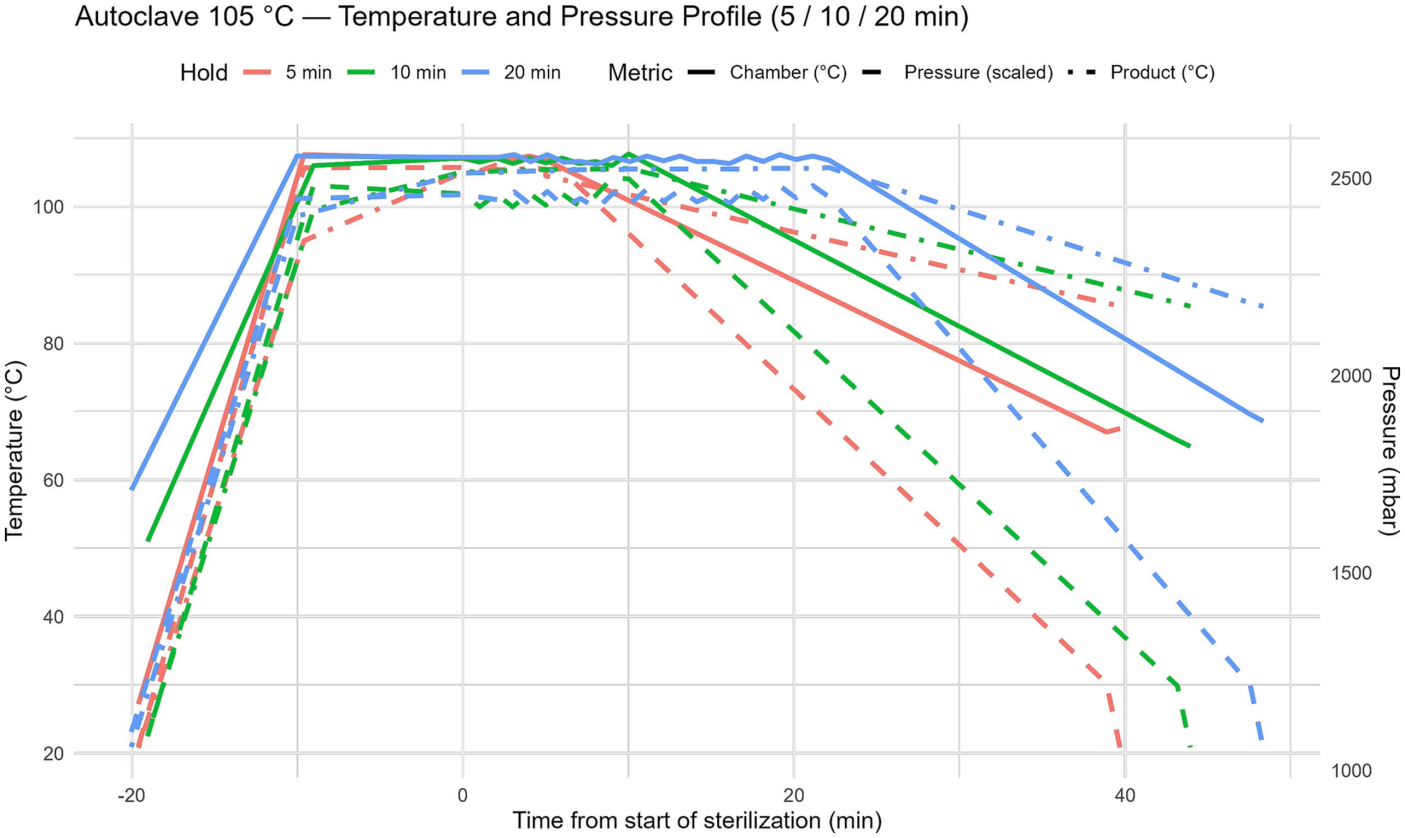
Autoclave processing profiles at 105 °C. Temperature and pressure dynamics during autoclaving at 105 °C with exposure times of 5, 10, and 20 min. The profiles illustrate the three key phases of thermal treatment: heating, holding at target temperature, and cooling.

**Figure 4:**
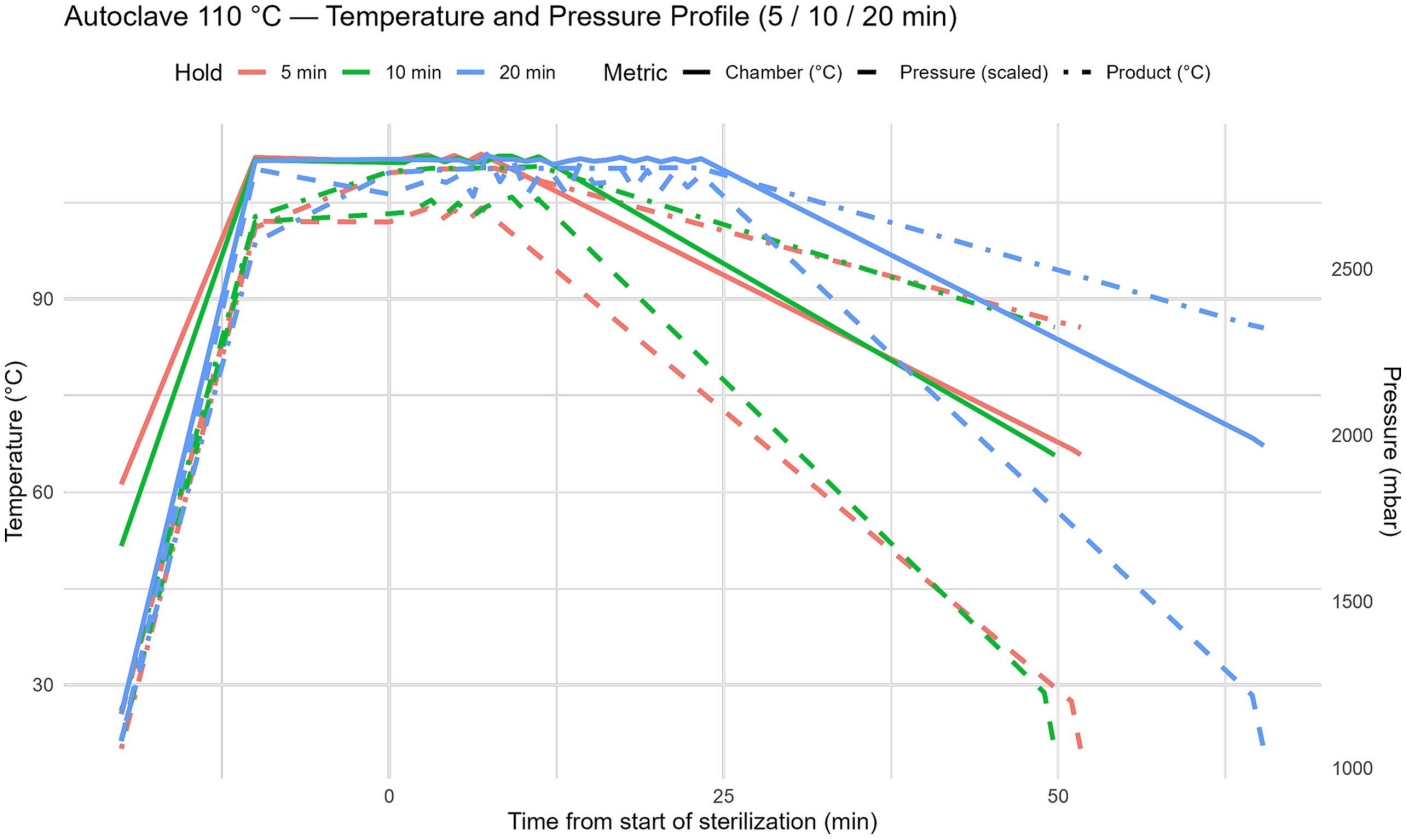
Autoclave processing profiles at 110 °C. Dynamics of temperature and pressure during autoclaving at 110 °C with exposure times of 5, 10, and 20 minutes. The profiles show the heating, holding, and cooling phases, highlighting the achievement and maintenance of stable temperature and pressure conditions throughout the treatment cycle.

**Figure 5:**
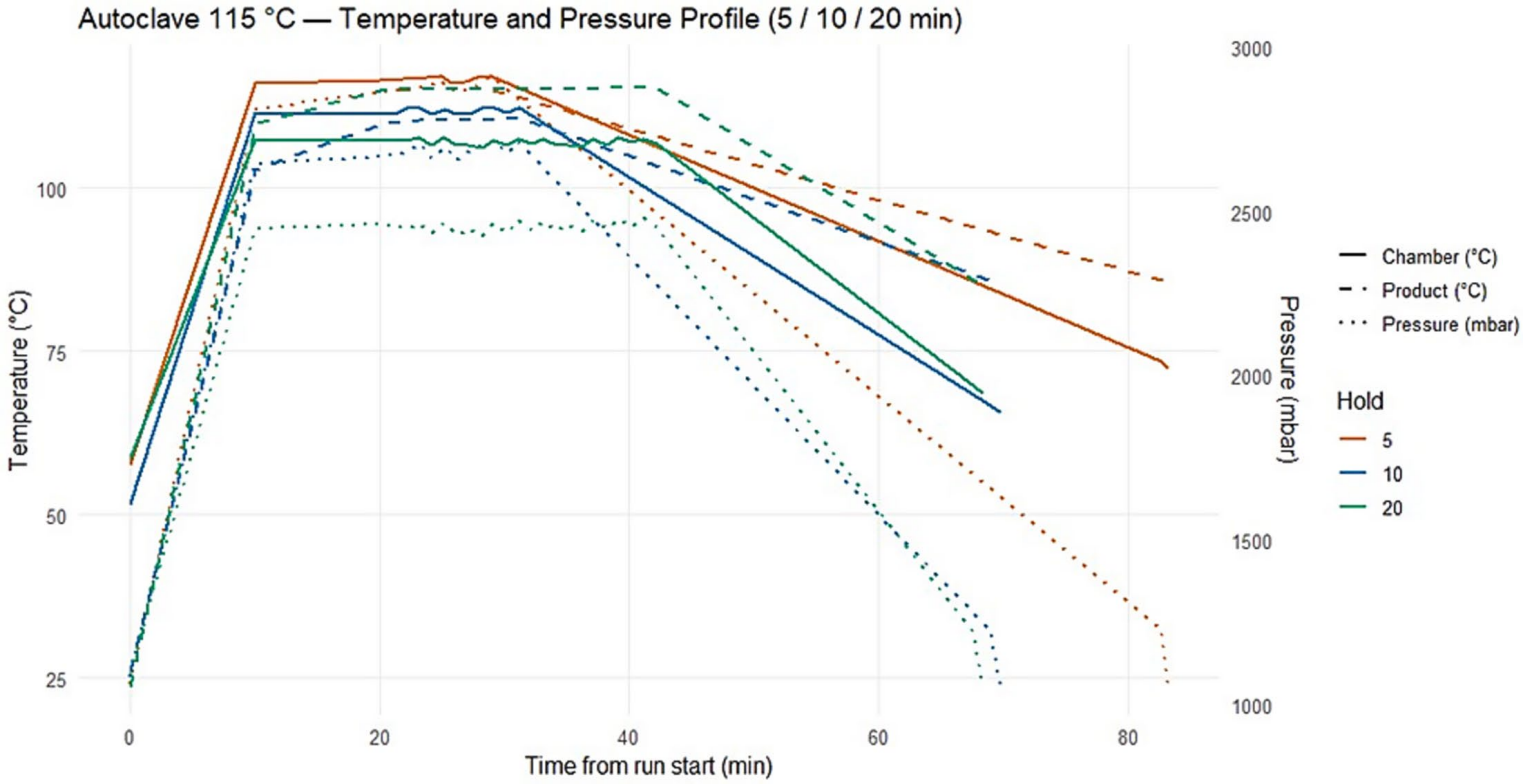
Autoclave processing profile at 115 °C/ Temperature and pressure dynamics during autoclaving at 115 °C with exposure times of 5, 10, and 20 min. These profiles represent the highest intensity treatment regimes in the study, illustrating the heating, holding, and cooling phases and the achievement of stable temperature and pressure conditions throughout the cycle.

Thermal treatment of the beverages was performed using a laboratory–scale autoclave under strictly controlled temperature and pressure conditions. Three moderate autoclaving regimes – 105 °C, 110 °C, and 115 °C – were evaluated, each applied for exposure times of 5, 10, and 20 min (Figure 2). These processing parameters were selected to represent practically relevant temperature–time combinations and to identify conditions that ensure microbiological safety and product stability without the addition of chemical preservatives. During each autoclaving cycle, dynamic temperature and pressure profiles were continuously recorded using the integrated data acquisition system of the autoclave. Temperature measurements included both chamber temperature and product temperature. The recorded profiles (Figures 3–5) illustrate three distinct phases of the thermal treatment process: (i) the heating phase, during which temperature gradually increased to the target level; (ii) the holding phase, in which the target temperature (105–115 °C) was maintained for the specified exposure time; and (iii) the cooling phase, characterized by a gradual reduction in both temperature and pressure.

Across all processing regimes, a stable temperature plateau was reached within 8–12 min, indicating uniform heat transfer and effective heating of the beverage matrix to the target temperature. Chamber pressure closely followed temperature changes, reaching approximately 1800–2,200 mbar at 105 °C, 2200–2,500 mbar at 110 °C, and up to 2,700 mbar at 115 °C. These temperature–pressure dynamics are critical for the effective inactivation of microorganisms, including heat–resistant spore–forming species.

The cooling phase was conducted in a controlled and gradual manner to minimize rapid physicochemical changes that could negatively affect protein stability, colloidal structure, and beverage texture.

### 2.3 Autoclaving conditions, storage temperatures and microbiological analyses

Figure 2 schematically illustrates the experimental design for autoclaving and microbiological evaluation. Autoclaving conditions (temperature–time combinations) were applied as described in Section 2.2. Pea protein isolate beverages were heat–treated in a laboratory autoclave (CFS–50H, TERPA Food–tech, Spain) at three temperatures (105 °C, 110 °C, and 115 °C), each combined with three exposure times (5, 10, and 20 min). For each temperature–time combination, nine samples (*n* = 9) were prepared.

After autoclaving, samples were divided into three subgroups (*n* = 3 per subgroup) to evaluate the effect of storage conditions on microbiological stability. The first subgroup was analysed immediately after thermal processing to determine the total microbial count, the presence of yeasts and molds, and the presence of *Escherichia coli*. The second subgroup was stored at 5 °C for 14 days, and the third subgroup was stored at 22 °C for the same period. After storage, identical microbiological analyses were performed for both stored subgroups.

pH values were measured at each experimental stage using a calibrated digital pH meter (SavenCompact S220, Saven Instrument, China). This experimental design enabled the simultaneous evaluation of the effects of thermal treatment intensity, storage temperature, and storage duration on the microbiological safety and stability of the final beverage products.

### 2.4 Microbiological evaluation of beverages

The microbiological quality of the beverages was assessed using internationally recognized laboratory methods in accordance with ISO standards and the microbiological criteria for food established by European Commission Regulation (EC) No. 2073/2005 (25) and the Regulations of the Cabinet of Ministers of the Republic of Latvia No. 461 (2014).

For analysis, 1 mL of each beverage sample was aseptically transferred to 9 mL of sterile distilled water to obtain a 10^−1^ dilution. The mixture was homogenized using a BagMixer® 400 (Interscience, France) to ensure uniform suspension. A 1 mL aliquot of this dilution was further diluted in 9 mL of sterile distilled water (10^−2^ dilution) and homogenized with an Ohaus Digital Vortex Mixer for 10 s prior to plating.

Microbial enumeration was performed using the spread plate method on appropriate media: total aerobic mesophilic counts were determined on Plate Count Agar (PCA) and incubated at 30 ± 1 °C for 72 ± 3 h according to ISO 4833–1:2013; yeasts and molds were enumerated on Potato Dextrose Agar (PDA) incubated at 25 ± 1 °C for five days following ISO 21527–1:2008; and *Escherichia coli* was selectively detected on ENDO agar incubated at 44 ± 0.5 °C for 24–48 h in accordance with ISO 16649–2:2001. After incubation, colonies were counted using a Scan® 500 automatic colony counter (Interscience, France), providing high–resolution imaging, accurate automatic colony recognition, and digital data acquisition. Results were expressed as colony–forming units per gram (CFU/g). Samples in which microbial counts were below the detection limit were recorded as <10 CFU/g. The obtained data were evaluated against current microbiological quality criteria for heat–treated food products.

### 2.5 Data processing, analysis, and visualization

Microbiological and pH data were processed, structured, and visualized using RStudio (R version 4.5.3, 2023). Data manipulation was performed using the tidyverse, dplyr, readxl, and tidyr packages, while visualizations were generated using ggplot2. Microbial counts were log-transformed (log₁₀ CFU/g) to enable comparison across different microorganism groups and processing regimes.

Data analysis was conducted using a descriptive approach without inferential statistical testing, as the study was designed to provide a descriptive evaluation of microbiological changes under different thermal treatment and storage conditions. Results were visualized using heatmap representations to illustrate trends across temperature–time combinations and storage conditions. Values below the detection limit were reported as <10 CFU/g and are indicated accordingly in the graphical representations.

Microbiological results were interpreted against established regulatory criteria (Cabinet of Ministers Regulations No. 461 (26); Regulation (EC) No. 2073/2005), which served as reference thresholds for assessing product safety and compliance. These thresholds were additionally indicated in the figures as reference lines to facilitate interpretation. The total aerobic microbial count (TAC) was compared with the quality criterion of ≤10^3^ CFU/g (3.0 log₁₀ CFU/g), while yeast and mold counts were interpreted using the quality limit of ≤50 CFU/g (1.7 log₁₀ CFU/g).

The presence of *Escherichia coli* was assessed based on the requirement that it must not be detected in finished products. The laboratory detection limit was <10 CFU/g; results below this threshold were recorded as “not detected.” For graphical representation only, these values were assigned a constant value of 1.0 log₁₀ CFU/g to allow visualization on a logarithmic scale. This approach was applied solely for visualization purposes and does not affect data interpretation or microbiological safety assessment.

This approach allowed evaluation of the combined effects of thermal treatment, storage temperature, and duration on the microbiological safety and stability of pea protein isolate beverages.

## 3 Results

### 3.1 Microbiological characterization of pea protein isolate beverage with blackcurrant and apple flavor (BAB)

The total aerobic microbial count (TAC) of the blackcurrant–apple pea protein isolate beverage (BAB) remained below the regulatory limit of ≤10^3^ CFU/g (3.0 log₁₀ CFU/g, Cabinet of Ministers Regulations No. 461) across all thermal treatment regimens (105–115 °C, 5–20 min). Immediately after thermal treatment, all samples showed microbial counts below the detection limit (<10 CFU/g), presented as 1.0 log₁₀ CFU/g in the figures for visualization purposes. After 14 days of storage at 22 °C, slightly higher TAC values were observed only in samples treated at 105 °C (5–20 min), reaching 1.8–2.0 log₁₀ CFU/g, while samples processed at 110 and 115 °C remained at the detection limit (1.0 log₁₀ CFU/g in figures). Storage at 5 °C was associated with minimal changes in TAC, with values remaining close to the detection limit in most cases, except for the 105 °C × 10 min treatment, where TAC reached 2.0 log₁₀ CFU/g, still below the regulatory threshold.Yeast and mold counts were below the detection limit (<10 CFU/g) immediately after thermal treatment across all regimes. After 14 days at 5 °C, only the 105 °C × 10 min treatment showed a minor increase (1.45 log₁₀ CFU/g), remaining below the regulatory limit of 50 CFU/g (1.7 log₁₀ CFU/g). At 22 °C, higher yeast and mold counts were observed in samples treated at 110 °C (1.9–2.1 log₁₀ CFU/g after 14 days), exceeding the permissible limit, whereas samples treated at 105 °C remained within or near the limit (1.0–1.2 log₁₀ CFU/g). Samples treated at 115 °C showed counts at the detection limit (≤1.0 log₁₀ CFU/g in figures) across all storage conditions. E*. coli* was not detected (<10 CFU/g) in any sample immediately after treatment or after storage at 5 °C or 22 °C, in accordance with regulatory requirements.

**Figure 6:**
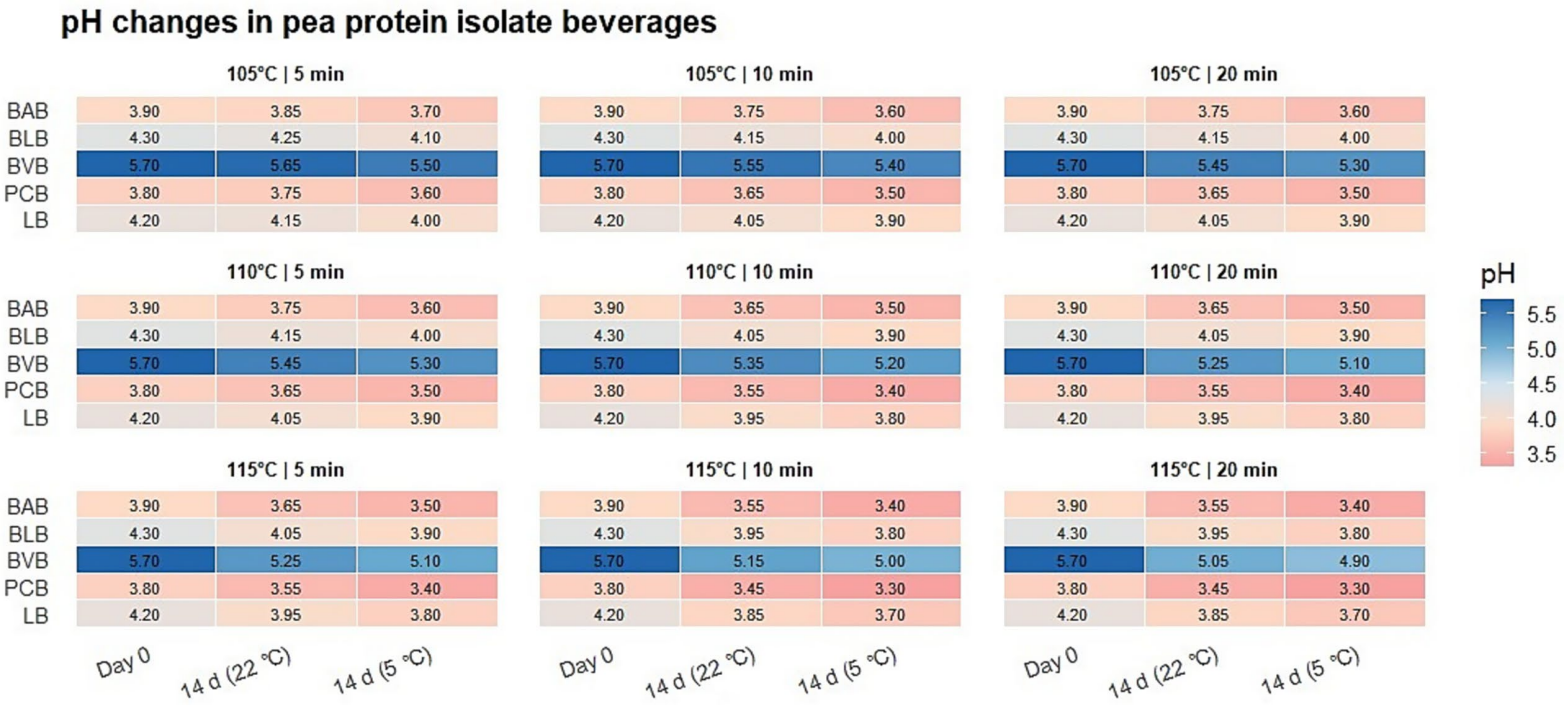
pH changes in pea protein isolate beverages. This figure presents pH changes in five pea protein isolate beverages (BAB, BLB, BVB, PCB, LB) under different autoclaving temperatures (105, 110, and 115 °C), processing times (5, 10, and 20 min), and storage conditions (immediately after processing and after 14 days at 5 °C and 22 °C).

**Figure 7:**
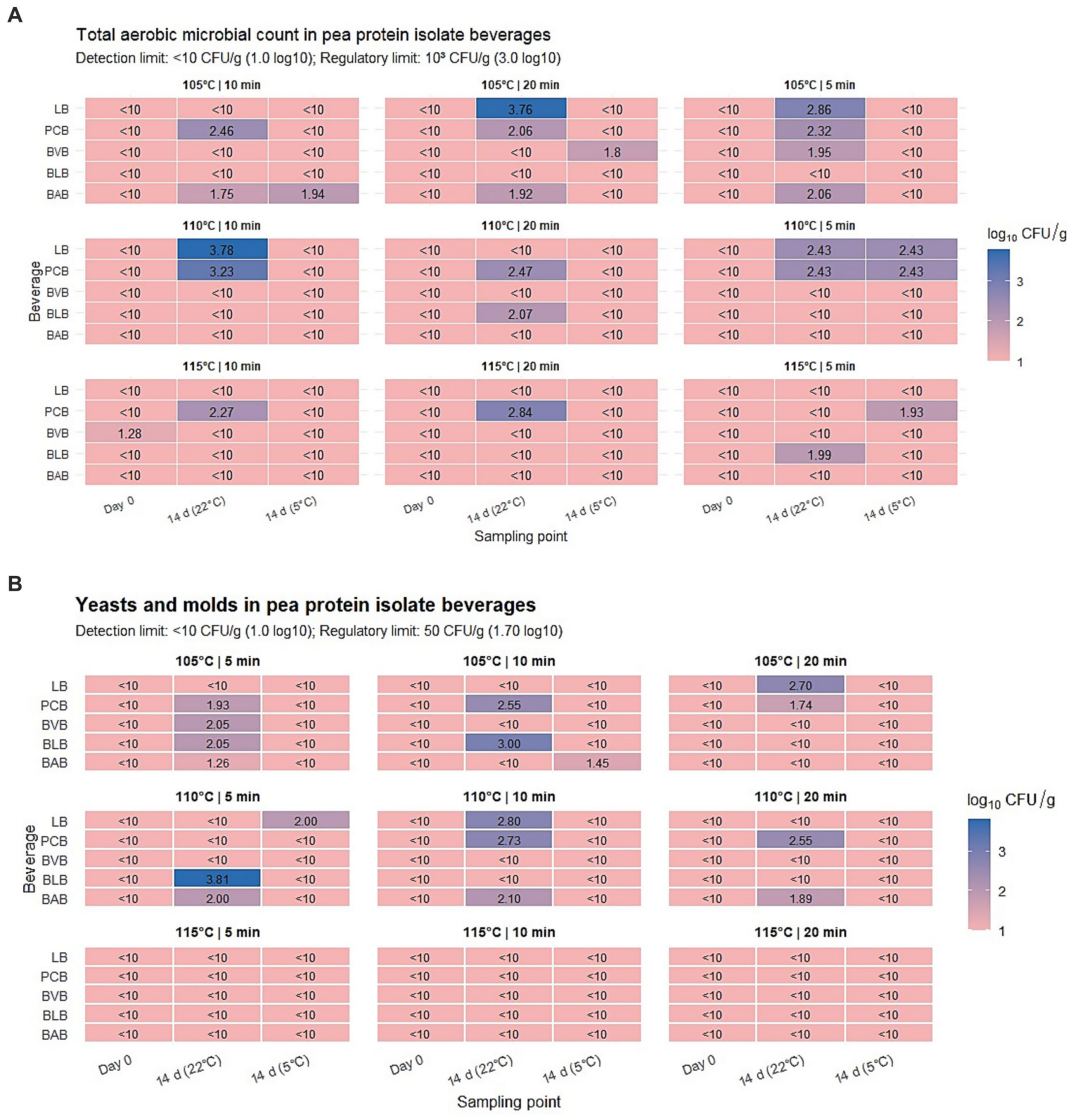
**(A)** Total aerobic microbial count in pea protein isolate all beverage. - BAB, BLB, BVB, PCB, LB. Heatmap representation of total aerobic microbial counts (log₁₀ CFU/g) in all pea protein isolate beverages (BAB, BLB, BVB, PCB, and LB) under different autoclaving regimes (105, 110, and 115 °C; 5, 10, and 20 min) and storage conditions (5 °C and 22 °C for 14 days). Values below the detection limit are indicated as <10 CFU/g (1.0 log₁₀), while the regulatory limit for total aerobic microorganisms is 10^3^ CFU/g (3.0 log₁₀). Color intensity reflects microbial load. **(B)** Yeasts and molds all beverages - BAB, BLB, BVB, PCB, LB. Heatmap representation of yeast and mold counts (log₁₀ CFU/g) in all pea protein isolate beverages (BAB, BLB, BVB, PCB, and LB) under different thermal treatment regimes (105, 110, and 115 °C, 5, 10, and 20 min) and storage conditions (5 °C and 22 °C for 14 days). Values below the detection limit are indicated as <10 CFU/g (1.0 log₁₀), and the regulatory limit for yeasts and molds is 50 CFU/g (1.7 log₁₀). Color intensity reflects microbial load.

The pH of the BAB beverage remained stable throughout all experiments, ranging from 3.4 to 3.8 (Figure 6). No notable differences related to temperature or processing time were observed, and fluctuations after 14 days of storage were minimal (<±0.05). Overall, the BAB beverage exhibited consistently low microbial counts under all tested conditions, with yeast and mold counts exceeding regulatory limits only in samples stored at 22 °C after treatment at 110 °C.(Figures 7A,B).

### 3.2 Microbiological characterization of pea protein isolate beverage with blueberry and lemon flavor (BLB)

The total aerobic microbial count (TAC) of the blueberry–lemon beverage (BLB) remained below the detection limit (<10 CFU/g), presented as 1.0 log₁₀ CFU/g in the figures, immediately after thermal treatment across all regimes. These values meet regulatory standards. After 14 days of storage at 22 °C, TAC remained low, with values ranging between 2.0–2.1 log₁₀ CFU/g at certain points, but never exceeding the regulatory limit of 3.0 log₁₀ CFU/g. Storage at 5 °C was associated with minimal changes, with TAC values remaining at the detection limit (1.0 log₁₀ CFU/g in figures). Yeast and mold counts were initially below the detection limit (<10 CFU/g), presented as 1.0 log₁₀ CFU/g in figures, for all thermal treatment regimes. After 14 days at 22 °C, higher values were observed in samples treated at 110 °C, particularly at 110 °C × 5 min, where counts reached above 3.0 log₁₀ CFU/g, exceeding the regulatory threshold of 50 CFU/g (1.7 log₁₀ CFU/g). Treatments at 105 °C showed lower counts, with values reaching ~2.0 log₁₀ CFU/g for the 5 min regime, while other treatments remained at the detection limit. All 115 °C treatments showed yeast and mold counts at ≤1.0 log₁₀ CFU/g, consistent across storage conditions. Storage at 5 °C was associated with minimal variation across all regimes. *E. coli* was not detected (<10 CFU/g) in any sample, both immediately after treatment and after 14 days at 5 °C and 22 °C, in accordance with regulatory requirements.

The pH of BLB remained stable across all thermal treatments and storage conditions, ranging from 3.8 to 4.2 (Figure 6). Fluctuations over time were minimal (<±0.05), with no notable differences observed between temperatures or storage duration (Figures 7A,B).

### 3.3 Microbiological characterization of pea protein isolate beverage with blueberry and vanilla flavor (BVB)

The total aerobic microbial count (TAC) of the blueberry–vanilla beverage (BVB) remained at low levels immediately after thermal treatment across all regimes, generally at 1.0 log₁₀ CFU/g. A slight increase to 1.2 log₁₀ CFU/g was observed for the 115 °C × 10 min treatment, remaining within acceptable microbiological limits. After 14 days of storage at 22 °C, TAC remained at 1.0 log₁₀ CFU/g for most treatments, with values reaching 1.9 log₁₀ CFU/g in the 105 °C × 10 min sample. Storage at 5 °C was associated with TAC values close to 1.0 log₁₀ CFU/g, except for the 105 °C × 20 min sample, where TAC reached 1.7 log₁₀ CFU/g. All values remained within regulatory limits under all storage conditions. Yeasts and molds were also below the detection limit (<10 CFU/g), presented as 1.0 log₁₀ CFU/g in figures, immediately after treatment across all regimes, reflecting negligible presence. After 14 days at 5 °C, yeast and mold counts remained at similar levels across all treatments. Storage at 22 °C was associated with higher values in the 105 °C × 5 min sample, reaching 1.9 log₁₀ CFU/g, slightly exceeding the recommended limit of 1.7 log₁₀ CFU/g. In all other treatments, yeast and mold counts remained near 1.0 log₁₀ CFU/g.

*E. coli* was not detected (<10 CFU/g) in any sample immediately after treatment or after storage at either 5 °C or 22 °C, in accordance with microbiological safety requirements. The pH of BVB was initially consistent at 5.7 across all thermal treatments (Figure 6). No notable differences related to temperature (105–115 °C) or exposure time (5–20 min) were observed immediately after treatment. Storage at 5 °C caused minor fluctuations (5.10–5.45), while storage at 22 °C resulted in lower pH values (4.9–5.3). Despite these changes, pH values remained within the acidic range across all conditions. (Figures 7A,B).

### 3.4 Microbiological characterization of pea protein isolate beverage with pomegranate and cranberry flavor (PCB)

Immediately after thermal treatment, the total aerobic microbial count (TAC) of the pomegranate–cranberry beverage (PCB) remained below the detection limit (<10 CFU/g), presented as 1.0 log₁₀ CFU/g in figures, across all regimes. These values are consistent with microbiological criteria for acidified beverages. Yeast and mold counts were also below the detection limit (<10 CFU/g), presented as 1.0 log₁₀ CFU/g in figures, and *Escherichia coli* was not detected (<10 CFU/g). After 14 days of storage at 22 °C, microbiological stability varied with thermal treatment Most samples remained at the detection limit level (1.0 log₁₀ CFU/g in figures); however, the sample treated at 105 °C × 5 min reached 2.8 log₁₀ CFU/g. Higher values were observed for 105 °C × 20 min and 110 °C × 10 min treatments, with TAC reaching 3.4–3.5 log₁₀ CFU/g. Yeast and mold counts showed a similar pattern, remaining at the detection limit level (1.0 log₁₀ CFU/g in figures) in most cases, with increases to 2.7–2.8 log₁₀ CFU/g in the same regimes. *E. coli* remained undetectable in all samples. Storage at 5 °C was associated with lower TAC values compared to 22 °C. TAC remained at the detection limit level (1.0 log₁₀ CFU/g in figures) in most samples, except for the 110 °C × 5 min treatment, where values reached 2.4 log₁₀ CFU/g. Yeast and mold counts remained undetectable in most refrigerated samples, except for the 110 °C × 5 min regime (2.0 log₁₀ CFU/g). *E. coli* remained below the detection limit in all refrigerated samples. The pH of PCB remained stable across all treatments and storage conditions (3.3–3.7) (Figure 6). Immediately after autoclaving, pH ranged 3.4–3.6, with minimal changes observed after 14 days at both 5 °C and 22 °C (Figures 7A,B).

### 3.5 Microbiological characterization of pea protein isolate beverage with lemon flavor (LB)

Immediately after thermal treatment, the total aerobic microbial count (TAC) of the lemon–flavoured beverage (LB) remained at 1.0 log₁₀ CFU/g across all temperature and time regimes, consistent with low initial microbial levels and regulatory standards. After 14 days of storage at 5 °C, TAC remained low in most treatments, with minor increases observed in 110 °C × 5 min and 115 °C × 10 min samples, all within acceptable limits. Storage at 22 °C was associated with higher TAC values, depending on the applied thermal treatment. The highest TAC values were observed in the 110 °C × 10 min sample (3.17 log₁₀ CFU/g) and the 115 °C × 20 min sample (2.85 log₁₀ CFU/g), while moderate values were recorded in 105 °C × 5 min (2.30 log₁₀ CFU/g), 105 °C × 10 min (2.45 log₁₀ CFU/g), and 115 °C × 10 min (2.25 log₁₀ CFU/g). At 22 °C, TAC values were higher compared to 5 °C across several treatments. Yeast and mold counts were below the detection limit (<10 CFU/g), presented as 1.0 log₁₀ CFU/g in figures, immediately after thermal treatment. Storage at 5 °C showed minimal variation over 14 days. At 22 °C, higher yeast and mold counts were observed, exceeding the acceptable limit in five thermal treatment regimes, particularly at 105 °C, 110 °C × 10 min, and 115 °C × 5 min, with values ranging from 1.9 to 2.7 log₁₀ CFU/g. *E. coli* was not detected (<10 CFU/g) in any sample. The initial pH of LB was 4.4, decreasing slightly to 4.2 immediately after autoclaving across all treatments (Figure 6). Storage at 5 °C resulted in minor decreases to 4.0–4.05, while storage at 22 °C showed lower values ranging from 3.7 to 3.9, with the lowest values observed in the 115 °C × 20 min samples. Across all conditions, pH values remained within the acidic range (Figures 7A,B).

## 4 Discussion

This study evaluated the microbiological and pH stability of five pea protein isolate beverages under different autoclaving regimens and two storage temperatures (+5 °C and +22 °C). The selected temperature range (105–115 °C) is consistent with literature recommendations for high-acid liquid products, where a pH below 4.6 is associated with reduced microbial heat resistance (27, 28).

High–acid products require lower thermal loads for microbial inactivation compared to low-acid foods, as pathogenic microorganisms exhibit reduced D-values under acidic conditions, allowing effective microbial control with moderate thermal treatment (29). Temperatures above 115–120 °C, in contrast, have been reported to be associated with protein denaturation, aggregation, and sediment formation, which may affect the stability and sensory properties of plant-based beverages (28, 30–32). Thermal treatment may also be associated with changes in the biological value of beverages due to protein denaturation and potential alterations in heat-sensitive micronutrients; however, these aspects were not evaluated in the present study. Given that the tested beverages were acidic (pH < 4.6), they are classified as low microbiological risk with respect to *Clostridium botulinum* toxin formation, eliminating the need for intensive sterilization (≥120 °C) typical of low–acid products (27). Therefore, the applied 105–115 °C range was considered technologically appropriate for achieving microbiological safety while maintaining product quality. Immediately after thermal treatment, all beverages demonstrated very low microbial counts, with total aerobic microorganisms and yeasts below the detection limit across all regimes. *E. coli* was not detected in any sample, consistent with literature describing reduced microbial survival in acidic environments under moderate thermal treatment (33).

During storage, differences in microbiological values were observed between storage temperatures and treatments, particularly at 22 °C. While total bacterial counts remained low, yeast and mold counts showed variation among beverages. These observations may be related to differences in composition, acidity, and ingredient types (fruit or herbal components).

Previous studies on plant-based beverages have reported changes in pH, acidity, and microbial populations during storage, particularly in fermented systems (34). Similar patterns of variation were observed in the present study, where changes in microbial counts were recorded under certain storage conditions. For example, the blackcurrant–apple beverage (BAB), containing blackcurrant juice/powder and apple juice, maintained a stable acidic environment throughout thermal treatment and storage. Total aerobic microorganisms remained low in all conditions, but yeast counts temporarily exceeded regulatory limits in samples stored at 22 °C after 110 °C treatment. In contrast, samples treated at 115 °C showed yeast counts within regulatory limits across storage conditions. These observations indicate that microbial counts remained low under most conditions, with higher values observed at 22 °C in specific treatments.

The blueberry–lemon beverage (BLB), formulated from blueberry and lemon juices, maintained a stable acidic environment throughout the study. The pH remained within the acidic range both immediately after thermal treatment and during storage at 5 °C and 22 °C. Microbiological analysis showed that total aerobic counts stayed within acceptable limits under all conditions; however, yeast and mold growth was sensitive to thermal treatment intensity. At 22 °C, higher yeast and mold counts were detected following lower-intensity treatments (105–110 °C), while treatments at 115 °C were associated with lower values. These findings align with reports describing the persistence of yeast-like microorganisms under certain storage conditions. The blueberry–vanilla beverage (BVB) differed from the other beverages in its higher pH, closer to neutral after thermal treatment, which gradually decreased during storage but remained higher than in the other beverages. Its composition, based on blueberry juice and vanilla extract, may be associated with lower acidity compared to the other formulations. While total aerobic counts remained low across all conditions, yeast and mold exceeded regulatory limits during storage at 22 °C after the mildest thermal treatment (105 °C × 5 min). Higher thermal treatments were associated with reduced microbial counts across storage conditions. These findings suggest that samples with higher pH showed higher microbial counts under certain conditions. The pomegranate–cranberry beverage (PCB) exhibited a strongly acidic environment due to the combined fruit juices, and its pH remained stable throughout all thermal treatment and storage conditions. Despite this, PCB was the most sensitive beverage in terms of microbial stability. Storage at 22 °C following 105–110 °C treatments corresponded with increases in both total microbial counts and yeast/mold counts exceeding regulatory limits. Conversely, samples processed at 115 °C maintained microbial levels within regulatory limits under all evaluated storage conditions. Overall, the results indicate that acidity plays a significant role in controlling microbial growth, although it did not fully offset the effects of lower-intensity thermal treatment under all conditions. Microbial counts varied across beverage compositions, pH levels, and thermal treatment regimes. The lemon beverage (LB) represented the simplest variant, with lemon juice as the sole source of acidity. Its pH remained within the acidic range throughout the study, with only slight decreases during storage at 22 °C. Microbiological analysis showed that total aerobic counts remained low across all conditions, while yeasts exceeded regulatory limits under several 105–110 °C treatments at 22 °C. Thermal treatment at 115 °C was associated with microbial counts remaining within regulatory limits across storage conditions. These findings suggest that, despite acidity, some beverages may show higher yeast counts under certain storage conditions. Overall, the microbiological stability of pea protein isolate beverages varied depending on the combination of pH, composition, and thermal treatment intensity. While acidic fruit beverages effectively inhibit bacterial growth, yeast–like microorganisms were observed to persist under certain storage conditions, particularly at elevated temperatures (21, 35). From a sustainable production perspective, longer thermal treatments (20 min) corresponded to higher processing intensity, although comparable microbiological outcomes were achieved in several cases using shorter treatment durations. Lower microbial counts were observed in samples treated at 110 °C for 10 min and 115 °C for 5–10 min across several conditions.

The pH of all beverages remained highly stable after thermal treatment and throughout storage, with fluctuations not exceeding 0.05–0.1 units, consistent with trends reported for acidic fruit-based beverages (22, 27, 36). These findings indicate that no substantial changes in acidity occurred during storage under the applied conditions. The results show that pea protein isolate can be used as a suitable raw material for beverage production, with microbiological stability observed under moderate thermal processing conditions (21, 37). Thermal processing conditions selected based on beverage composition and acidity were associated with microbiological stability while maintaining moderate processing intensity. The addition of vitamins and minerals, including magnesium and B vitamins, showed no clear association with changes in microbiological counts under the studied conditions. While minerals can act as cofactors in microbial metabolism and vitamins as growth factors, their concentrations in functional beverages are typically not associated with substantial microbial growth under acidic conditions. Microbiological stability in these beverages has been described as being influenced by multiple factors, including pH, water activity, nutrient availability, storage temperature, and thermal treatment intensity (15, 16). Organic acids such as ascorbic acid contribute to pH regulation and oxidative stability but are not considered strong antimicrobial preservatives on their own; therefore, yeast and mold growth may still occur under certain storage conditions (13, 21).

In conclusion, pea protein isolate beverages with acidic pH maintained microbiological stability under moderate thermal processing and refrigerated storage conditions, while higher microbial counts were observed under certain room temperature conditions. These findings support the use of moderate thermal treatment combined with acidic pH to produce microbiologically stable beverages without preservatives.

### 4.1 Limitations of the study

This study provides important insights into the microbiological and chemical stability of pea protein isolate beverages; however, several limitations should be considered. First, the beverages analysed were formulated without synthetic preservatives in line with the clean–label approach. While this reflects current industry trends, it increases sensitivity to microbial changes during storage, particularly at elevated temperatures. Consequently, the results reflect the behaviour of preservative–free systems and cannot be directly extrapolated to similar products containing preservatives. Second, the study evaluated stability over 14 days, which allows for assessment of short–term microbiological and chemical behaviour but does not capture long–term storage effects. Yeast and mold growth, as well as other spoilage processes, may occur over extended periods beyond this timeframe. Third, microbial evaluation focused on total aerobic microorganisms, yeasts, molds, and *Escherichia coli*. Third, microbial evaluation focused on total aerobic microorganisms, yeasts, molds, and *Escherichia coli*. While these are standard indicators for food safety, they do not allow identification of specific species or strains responsible for observed changes. Detailed microbial profiling could provide deeper insights into spoilage mechanisms. Additionally, the inclusion of specific pathogens such as *Salmonella* spp. and *Listeria monocytogenes*, as well as spore-forming bacteria, viable but non-culturable (VBNC) cells, and lactic acid bacteria (LAB), could enable a more comprehensive assessment of microbiological safety and stability in future studies. While these are standard indicators for food safety, they do not allow identification of specific species or strains responsible for observed changes. Detailed microbial profiling could provide deeper insights into spoilage mechanisms. Fourth, water activity is a key factor influencing microbial growth. Although the high moisture content of the beverages suggests a high–water activity, direct measurement would allow more precise interpretation of microbiological stability. Fifth, the beverages included multiple functional ingredients – protein isolate, fruit juices, fibre, vitamins, and minerals –whose interactions may influence microbial behaviour. The study did not isolate the effects of individual components, so conclusions pertain to the overall system rather than specific ingredients. Sixth, experiments were conducted under controlled laboratory conditions. In industrial production, additional factors such as packaging type, oxygen permeability, and scale–up variations may influence beverage stability and microbiological quality. Despite these limitations, the study provides valuable guidance for optimizing thermal processing of pea protein–based beverages under clean–label conditions, offering a foundation for future investigations under extended storage and industrial settings. Future studies should include a larger number of samples and extended storage periods to further enhance the robustness and applicability of the results.

## 5 Conclusion

All pea protein isolate beverages analysed in this study were microbiologically safe immediately after thermal treatment, with total aerobic microbial counts, yeasts, and molds at or near the detection limit, and *Escherichia coli* not detected in any sample. Storage temperature was associated with differences in microbiological stability, with refrigerated storage at 5 °C maintaining low microbial counts across all beverage variants, whereas storage at 22 °C was associated with increased yeast and mold counts in several compositions. Yeasts and molds were identified as the primary microbiological limiting factor, particularly under room–temperature storage and following lower or intermediate thermal treatment regimens (105–110 °C). The intensity of thermal treatment was closely associated with beverage composition and pH. Beverages with lower pH exhibited reduced microbial counts under the studied conditions, while those with higher pH or less acidic matrices were associated with increased microbial counts during storage. Among the thermal regimes tested, autoclaving at 115 °C maintained microbial counts within regulatory limits across all beverages, representing a condition under which stable microbiological profiles were consistently observed.

The results indicate that pea protein isolate beverages can maintain microbiological stability under moderate thermal treatment conditions without the addition of chemical preservatives. These findings are consistent with the production of plant-based beverages exhibiting stable microbiological profiles through combination of acidic pH and thermal processing.

## Data Availability

The original contributions presented in the study are included in the article/supplementary material, further inquiries can be directed to the corresponding author/s.
